# Label-Free Optical Metabolic Imaging in Cells and Tissues

**DOI:** 10.1146/annurev-bioeng-071516-044730

**Published:** 2023-04-27

**Authors:** Irene Georgakoudi, Kyle P. Quinn

**Affiliations:** 1 Department of Biomedical Engineering, Tufts University, Medford, Massachusetts, USA;; 2 Genetics, Molecular and Cellular Biology Program, Graduate School of Biomedical Sciences, Tufts University, Boston, Massachusetts, USA; 3 Department of Biomedical Engineering and the Arkansas Integrative Metabolic Research Center, University of Arkansas, Fayetteville, Arkansas, USA

**Keywords:** metabolic imaging, optical metabolic imaging, redox, NADH, FAD, autofluorescence, fluorescence lifetime, glycolysis, oxidative phosphorylation, multiphoton microscopy, two-photon excited fluorescence

## Abstract

Over the last half century, the autofluorescence of the metabolic cofactors NADH (reduced nicotinamide adenine dinucleotide) and FAD (flavin adenine dinucleotide) has been quantified in a variety of cell types and disease states. With the spread of nonlinear optical microscopy techniques in biomedical research, NADH and FAD imaging has offered an attractive solution to noninvasively monitor cell and tissue status and elucidate dynamic changes in cell or tissue metabolism. Various tools and methods to measure the temporal, spectral, and spatial properties of NADH and FAD autofluorescence have been developed. Specifically, an optical redox ratio of cofactor fluorescence intensities and NADH fluorescence lifetime parameters have been used in numerous applications, but significant work remains to mature this technology for understanding dynamic changes in metabolism. This article describes the current understanding of our optical sensitivity to different metabolic pathways and highlights current challenges in the field. Recent progress in addressing these challenges and acquiring more quantitative information in faster and more metabolically relevant formats is also discussed.

## OVERVIEW OF METABOLIC IMAGING MODALITIES

Metabolic research encompasses a broad range of investigations involving different chemical reactions that convert molecules to energy or other building blocks necessary to assemble proteins, lipids, nucleic acids, and carbohydrates. This work can focus on the level of the whole organism, specific tissues, individual cells, or individual organelles, such as mitochondria. A variety of tools, such as respirometry, have been developed to evaluate aspects of metabolism at different spatial and temporal scales (1–4). Among the most impactful and flexible tools at our disposal to characterize metabolism are those involving optical imaging and spectroscopy. Optical imaging, in particular, has become an indispensable part of biomedical research to provide functional, morphological, and compositional details of cells, tissues, and organs.

Metabolic imaging at the whole-body or tissue level can be achieved though clinical imaging modalities that rely on endogenous or exogenous sources of contrast (Table 1). Positron emission tomography and single-photon emission computed tomography allow for imaging of metabolism in the body through exogenous radionuclide-based contrast agents (5, 6). Magnetic resonance imaging (MRI) can provide structural information, and blood-oxygen-level dependent imaging can further allow for assessments of oxygen consumption within tissues (7). Magnetic resonance spectroscopy can also be used as a complement to MRI to evaluate the presence of different metabolites and lipids within specific regions of interest in tissues (8, 9). However, a variety of lower cost optical techniques have been developed to characterize metabolism at finer levels of temporal or spatial resolution.

Optical imaging modalities that rely on endogenous sources of contrast to characterize metabolism take advantage of a variety of sources of contrast, including absorption, scattering, and fluorescence (Table 1). Absorption-based contrast can be obtained from oxygenated and deoxygenated hemoglobin, lipids, and water by interrogating tissue using wavelengths of light ranging from the visible to mid-infrared bands (10–12). Extensive work to characterize hemoglobin content and oxygen saturation has been performed on a variety of tissues and disease states using multispectral imaging modalities such as diffuse optical tomography (13), diffuse reflectance spectroscopy (14), optical coherence tomography (OCT) (15, 16), and photoacoustic imaging (11). Through these different modalities, vascular and tissue oxygenation can be mapped at a variety of spatial scales, from tens of microns to centimeters.

At the cellular and subcellular scale, a variety of microscopy techniques are available to interrogate different aspects of metabolism. Exogenous fluorophores have been designed or used to probe different aspects of metabolism using standard scientific microscopes. Of note, TMRE (tetramethylrhodamine, ethyl ester) is often used to fluorescently label active mitochondria and measure membrane potential (17). Glucose uptake within cells and tissues can be visualized through analogs such as fluorescently labeled 2-deoxyglucose (18). Cellular reactive oxygen species (ROS) are commonly assessed through fluorescence microscopy using DCFDA, CellROX^™^, or MitoSOX^™^ kits (19, 20). A variety of fluorescent labels have also been developed for measuring cellular redox state as well (21–23). More generally, different mitochondrial dyes can be used to visualize mitochondrial organization and observe their fusion and fission dynamics. However, probing metabolism with any of these exogenous labels can alter cellular bioenergetics and makes longitudinal assessments of dynamic metabolic changes challenging.

Label-free microscopy techniques that rely on endogenous sources of contrast are well suited for quantitative assessments of living cells and organisms. The use of light to noninvasively investigate aspects of cellular function without interfering with a specimen’s physiology is a major advantage in metabolic research (Table 1). In particular, multiphoton microscopy (MPM) offers a set of techniques that have been used for probing different aspects of metabolism through two-photon excited fluorescence (TPEF) and coherent Raman imaging techniques, such as stimulated Raman scattering (SRS) and coherent anti-Stokes Raman scattering (CARS). MPM takes advantage of nonlinear light matter interactions that generally occur only at the focal plane, which provides intrinsic depth sectioning. Through the use of near-infrared light and more efficient collection optics, MPM enables deeper and less damaging 3D imaging than similar laser scanning techniques, such as confocal microscopy. Over the last 15 years, CARS and SRS have been used to monitor intracellular lipid metabolism and storage in a variety of cell and tissue types (24–26). However, the majority of MPM-based investigations of cellular metabolism focus on the imaging of reduced nicotinamide adenine dinucleotide (NADH) and flavin adenine dinucleotide (FAD) through TPEF.

NADH and FAD autofluorescence was originally characterized in the middle of the twentieth century by Britton Chance and colleagues (27–29). Because these coenzymes are key to linking the catabolism of carbon molecules to adenosine triphosphate (ATP) production through oxidative phosphorylation, NADH and FAD imaging has been used to study metabolism noninvasively in a variety of applications in the decades that followed. A number of reviews over the last 10 years have highlighted the growth and increasing breadth of NADH and FAD autofluorescence imaging since the advent of MPM (30–33). Rather than surveying these applications, this review evaluates how NADH and FAD are sensitive to different metabolic pathways and key physiological and cellular events. Challenges in NADH and FAD imaging are also covered, including the potential effects of other light–matter interactions and safety considerations. Finally, we cover recent progress in addressing these challenges, as well as efforts to mature the technology and acquire more quantitative autofluorescence information in faster and more meaningful formats.

## METABOLIC PATHWAYS INVOLVING NAD(P)H AND FAD

**Figure 1:**
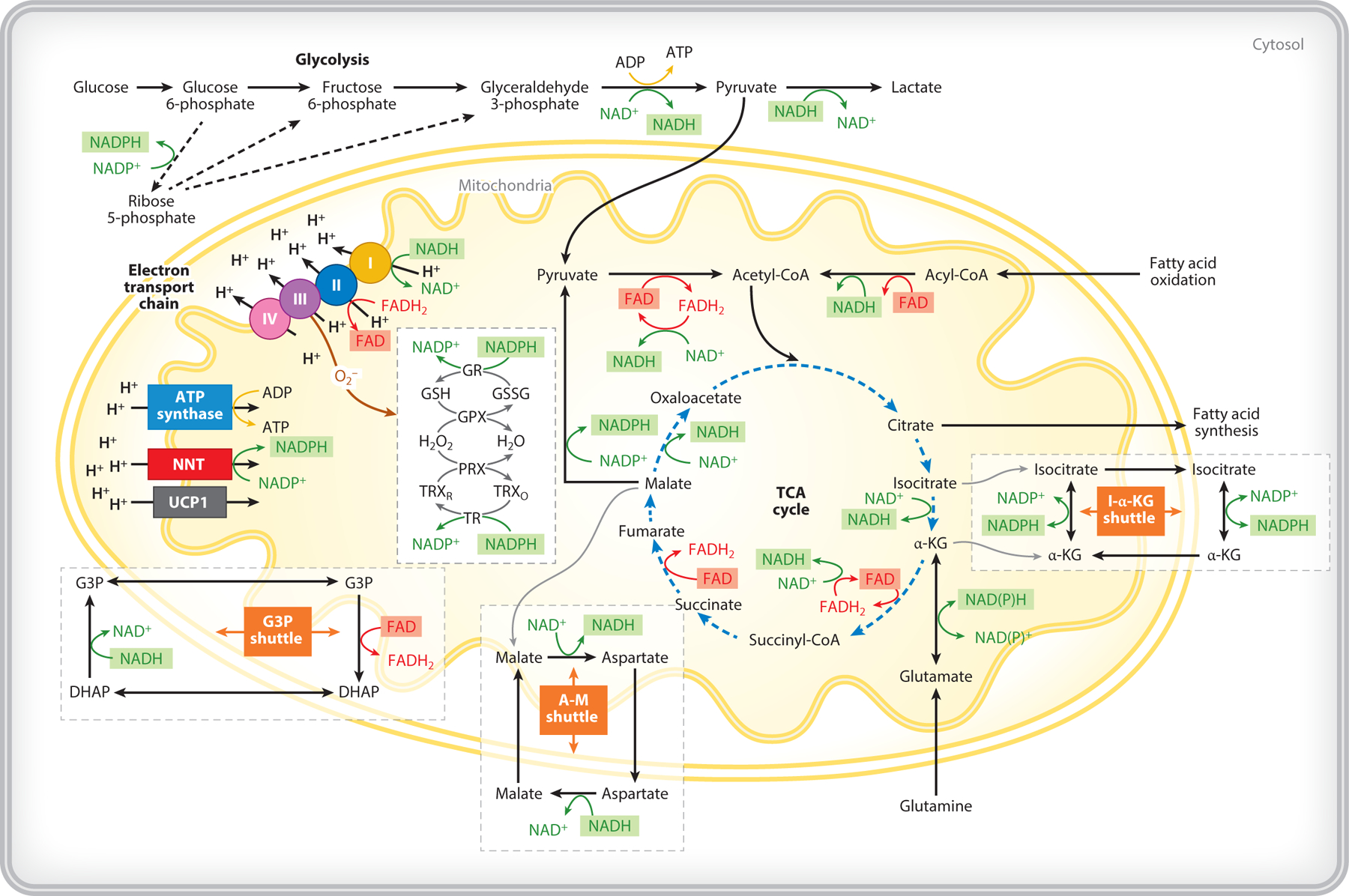
Overview of key cellular metabolic pathways in which NAD(P)H and FAD are involved, including glycolysis, the TCA cycle, oxidative phosphorylation, fatty acid oxidation and synthesis, and the glutathione pathway. Important shuttles such as the G3P, A-M, and I-α-KG shuttles, responsible for maintaining distinct redox states in the mitochondria and the cytosol, are also included. Abbreviations: A-M, aspartate–malate; CoA, coenzyme A; FAD, flavin adenine dinucleotide; G3P, glycerol-3-phosphate; GSH, glutathione; I-α-KG, isocitrate-α-ketoglutarate; NAD^+^, nicotinamide adenine dinucleotide; NADH, reduced nicotinamide adenine dinucleotide; NADP^+^, nicotinamide adenine dinucleotide phosphate; NADPH, reduced nicotinamide adenine dinucleotide phosphate; TCA, tricarboxylic acid. Illustration created by Adriana Sanchez Hernandez, Department of Biomedical Engineering, Tufts University.

Metabolism involves a complex set of cellular pathways responsible for biosynthesis and energy production (Figure 1). Glycolysis and oxidative phosphorylation are two key metabolic pathways that contribute to the overall cellular metabolic state. Glycolysis takes place in the cytosol and involves the breakdown of glucose to pyruvate, during which ATP is produced, and NAD^+^ is reduced to NADH. Along the way, a number of intermediate molecules (i.e., metabolites) that are important for biosynthesis are produced as well. Pyruvate can be converted to lactate to oxidize NADH to NAD^+^, or it can be transferred into mitochondria. Once in the mitochondria, more NAD^+^ is reduced to NADH as pyruvate is broken down into acetyl coenzyme A (acetyl-CoA) for use in fueling the tricarboxylic acid (TCA) cycle. The TCA cycle produces additional NADH as carbon molecules are further catabolized to CO_2_. The energy carried by NADH in the form of electrons is transferred to the protein complexes of the electron transport chain (ETC) along the inner mitochondrial membrane. These complexes transfer the electrons received from NADH in a series of steps that produce a proton gradient across the mitochondrial membrane with oxygen serving as the final electron acceptor. This proton gradient ultimately powers the efficient production of ATP, the energy currency of the cell.

Acetyl-CoA can also be produced from β oxidation of fatty acids that yields NADH and FADH_2_, which can be oxidized by complex I and complex II of the ETC, respectively. Glutamine can also enter the mitochondria and serve as a fuel for the TCA cycle after it gets converted to glutamate and α-ketoglutarate. Fatty acid synthesis, on the other hand, requires citrate (synthesized in the TCA) to exit mitochondria, thus, draining the TCA of carbon that could otherwise be catabolized down to CO_2_ for efficient ATP production.

The NAD^+^/NADH ratio is highly compartmentalized and tightly controlled to maintain homeostasis (34, 35). In the cytosol, NADH is produced during glycolysis. In the mitochondria, NADH is produced in the TCA cycle and fatty acid oxidation. Unlike the outer mitochondrial membrane, the inner mitochondrial membrane is not permeable to NADH. Thus, shuttles, including the aspartate–malate shuttle and the glycerol-3-phosphate (G3P) shuttle, are utilized to maintain distinct NAD^+^/NADH ratios within the cytosol and the mitochondria (34). For example, conversion of aspartate to malate in the cytosol involves the oxidation of NADH to NAD^+^, while the malate produced in the cytosol is transferred into the mitochondria by the aspartate–malate shuttle, leading to conversion of malate to aspartate and reduction of NAD^+^ to NADH. This shuttle can operate in both directions. The G3P shuttle, on the other hand, is unidirectional and involves the oxidation of NADH to NAD^+^ and generation of G3P in the cytosol, which is transported to the mitochondria and used to drive the reduction of FAD to FADH_2_, which, as mentioned above, is used by complex II. Under physiologic conditions, the cytosolic NAD^+^/NADH ratio is significantly higher than that of the mitochondrial pool, as NAD^+^ is required to drive glycolysis (for example, the ratio is ~700:1 in the cytosol versus ~7–8:1 in the mitochondria of rat hepatocytes) (35). Thus, these shuttles play an important role in maintaining these highly distinct redox pools, especially when the mitochondrial membrane potential generation is uncoupled from ATP production.

Given that oxygen is the final acceptor of electrons in the ETC, the production of reactive oxygen species, such as superoxide, is an unavoidable consequence of oxidative phosphorylation. Thus, it is critical for cell function to have robust antioxidant defenses (34–36). NADPH is a key biomolecule in the maintenance of the glutathione (GSH) peroxidase and peroxiredoxin antioxidant systems in the mitochondria. NADPH can be produced from NADH in the mitochondria through nicotinamide nucleotide transhydrogenase, but it can also be produced through a variety of pathways, including different mitochondrial isozymes. In the cytosol, NADPH is primarily generated from nicotinamide adenine dinucleotide phosphate (NADP^+^) in the pentose phosphate pathway, and it is a critical component for the synthesis of nucleotides, amino acids, and lipids. Under physiological conditions, more than 95% of mitochondrial NADP^+^ is reduced as the protons of the molecule are used for GSH generation. So, while NADH utilization by the ETC leads to ROS production, NADPH is typically used to mitigate the cytotoxic effects of ROS. Nevertheless, excess accumulation of glutathione or NADPH leads to reductive stress and also contributes to generation of oxygen radicals and superoxide (34). Thus, maintenance of NADP^+^/NADPH pools is also tightly controlled. The inner mitochondrial membrane is impermeable to NADPH; another shuttle, the isocitrate-α-ketoglutarate shuttle, controls communication between cytosolic and mitochondrial NADP^+^/NADPH pools.

Beyond complex biochemical machinery and control mechanisms, mitochondria have evolved rapid and transient morphological adaptations to optimize metabolic function (37). Mitochondria undergo fusion and fission throughout the life cycle of a cell to enhance energy production and delivery, ensure mitochondrial quality control, and participate in events that are considered crucial for programmed cell death, innate immunity, and autophagy (37). Fused mitochondrial networks are typically observed within cells that rely on oxidative phosphorylation for energy production (38); however, they have also been associated with cell survival and protection from autophagy during nutrient starvation (39). Both enhancement (40) and degradation (41) of mitochondrial networks have been reported following interventions that lead to life extension. While fragmented mitochondria are naturally more prevalent in dividing and moving cells (i.e., cells with high glycolytic rates), abnormal mitochondrial fragmentation is often associated with stress and/or disease (e.g., neurodegenerative diseases) (42).

The metabolic pathways we discuss in this brief overview represent only a small fraction of the cellular networks that are responsible for biosynthesis and energy production. However, it is clear that spatial and temporal control of NAD(P)^+^/NAD(P)H pools plays a central role in metabolic function and that such pools are highly sensitive to changes that may occur during normal aging and the development or evolution of disease or in response to environmental insults or treatments [NAD(P)^+^ and NAD(P)H are used to refer to both the phosphorylated and unphosphorylated moieties of the fluorophores]. Thus, methods that enable us to monitor NAD(P)^+^/NAD(P)H levels within cells and mitochondria in a noninvasive, sensitive, specific, and quantitative manner in physiologically relevant specimens have the potential to make a broad and significant biomedical impact.

## OVERVIEW OF NAD(P)H AND FAD AUTOFLUORESCENCE

None other than Otto Warburg first reported the 340-nm absorption band associated with diphosphopyridine nucleotide (DPNH), which is how NADH was referred to in the original literature. It was, of course, the pioneering work of Britton Chance and his colleagues that established the autofluorescence excitation and emission properties of NADH. A series of intense studies established its use to assess redox state and mitochondrial function in a wide range of samples, from isolated mitochondria to in vivo tissues (29). However, the presence of the main NADH fluorescence excitation peak in the UV poses significant penetration depth and cytotoxicity limitations, particularly for live cell or tissue imaging. The advent of nonlinear (two- and three-photon) excited fluorescence microscopy has enabled multiparametric high-resolution metabolic function imaging of tissues over depths that extend several hundred microns, sufficient to gain unique insights that promise to improve our understanding of the role of different metabolic pathways in development, aging, and numerous diseases.

**Figure 2:**
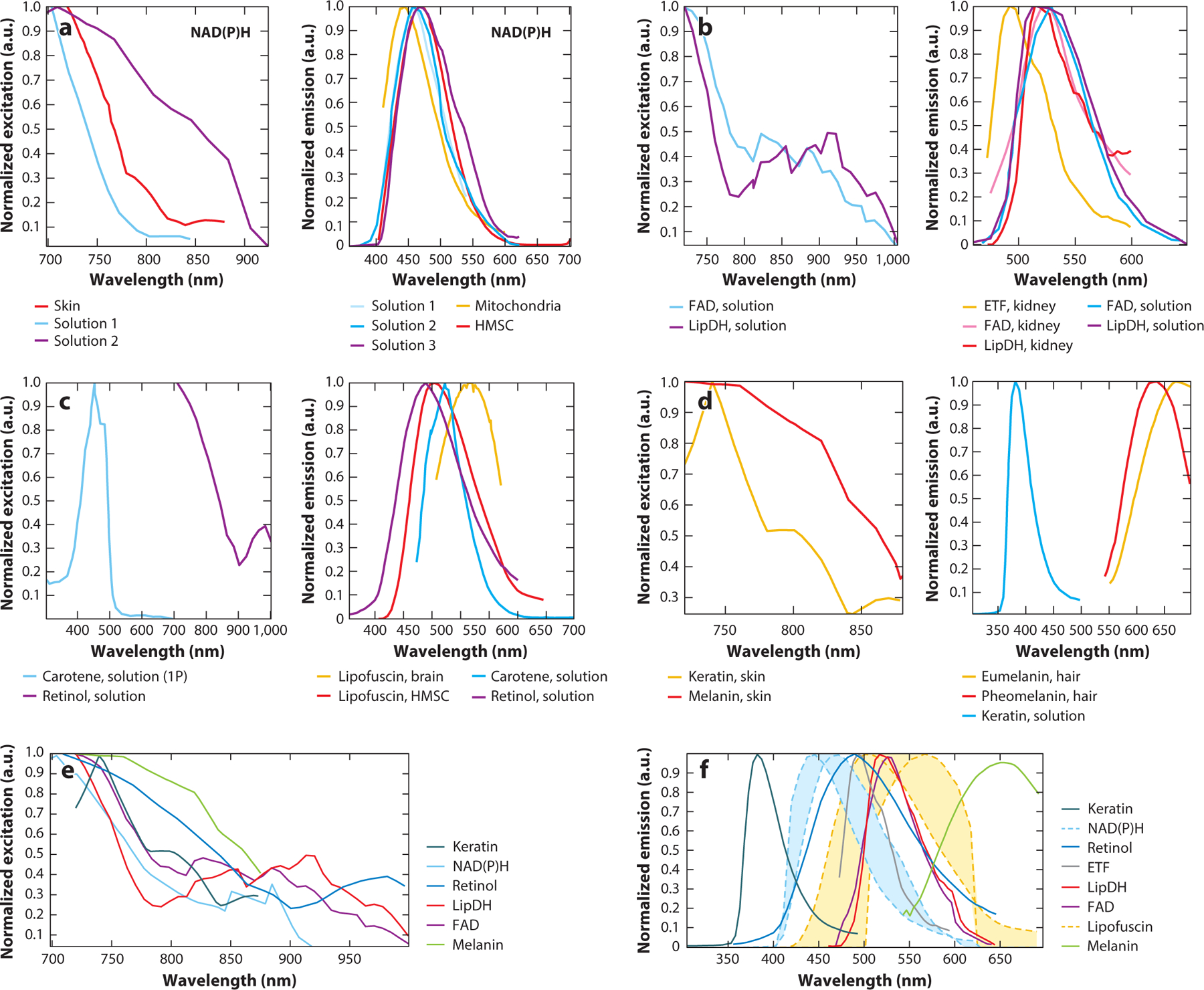
Representative excitation/action cross-section (*left*) and emission (*right*) spectra from key fluorophores, reported by various investigators, highlighting similarities and differences among different studies. Spectra were generated using the WebPlotDigitizer v.4.5 app and reproduced from the original publications. 1P spectra are included when two-photon spectra were not available. Unless otherwise noted, excitation spectra are two-photon action cross sections. (*a*, *left*) NADH excitation spectra. Skin spectra adapted with permission from Reference 80, copyright 2010 The Optical Society; solution 1 spectra adapted with permission from Reference 43; and solution 2 spectra adapted with permission from Reference 111, copyright 2003 National Academy of Sciences. (*a*, *right*) NADH emission spectra. Mitochondria spectra adapted with permission from Reference 44; HMSC spectra adapted with permission from Reference 50; solution 1 spectra adapted with permission from Reference 44; solution 2 spectra adapted with permission from Reference 43; and solution 3 spectra adapted with permission from Reference 111, copyright 2003 National Academy of Sciences. (*b*, *left*) FAD and associated flavoprotein excitation spectra. Spectra adapted with permission from Reference 43. (*b*, *right*) FAD and associated flavoprotein emission spectra. ETF, FAD, and LipDH kidney spectra adapted with permission from Reference 58, copyright 1993 Elsevier; FAD and LipDH solution spectra adapted with permission from Reference 43. (*c*, *left*) Carotenoid and retinol lipofuscin excitation spectra. Carotene spectra adapted from Reference 70 (CC BY 4.0); retinol spectra adapted with permission from Reference 111, copyright 2003 National Academy of Sciences. (*c*, *right*) Lipofuscin, carotene, and retinol emission spectra. Lipofuscin, brain spectra adapted from Reference 70 (CC BY 4.0); lipofuscin, HMSC spectra adapted with permission from Reference 50; carotene spectra adapted from Reference 70 (CC BY 4.0); and retinol spectra adapted with permission from Reference 111, copyright 2003 National Academy of Sciences. (*d*, *left*) Keratin/melanin excitation spectra. Spectra adapted with permission from Reference 80, copyright 2010 The Optical Society. (*d*, *right*) Eumelanin, pheomelanin, and keratin emission spectra. Eumelanin and pheomelanin spectra adapted from Reference 82 (CC BY 4.0); keratin spectra adapted with permission from Reference 85, copyright 2005 The Optical Society. (*e*, *f*) Compilation of all fluorescence excitation and emission spectra. For NADH and lipofuscin, the dotted lines encompass all corresponding spectra, while the shaded areas highlight the range of variation. Legends show the fluorophore and the associated reference from which spectral data were extracted. Marker spacing was chosen arbitrarily and does not reflect the number of points collected in the original data. Intensities are normalized to the maximum value reported. Abbreviations: 1P, one photon; ETF, electron transfer flavoprotein; FAD, flavin adenine dinucleotide; HMSC, human mesenchymal stem cell; LipDH, lipoamide dehydrogenase; NADH, reduced nicotinamide adenine dinucleotide; NADPH, reduced nicotinamide adenine dinucleotide phosphate. Figure generated by Varshini Ramanathan, Tufts University.

TPEF involves the simultaneous absorption of two photons, each of half the energy (and twice the wavelength), to bring molecules to an excited state. In practice, detectable TPEF occurs only at the focal plane of an objective lens where the photon density is highest, and this enables high-resolution images of cells and tissues in 3D through raster scanning. The two-photon excitation action cross-section spectrum of NAD(P)H is consistent with what would be expected after doubling the wavelength of the single-photon excitation spectrum (at least in the 700- to 850-nm range) (43) (Figure 2). The reported TPEF emission of endogenous fluorophores is highly consistent with single-photon excited fluorescence characteristics. NADH and NADPH excitation and emission spectra overlap highly; for this reason, NAD(P)H is often used to refer to the combined signal from the two fluorophores (43). Peak emission of NAD(P)H in solution is typically between 460 and 470 nm, while binding to substrates, such as complex I or several dehydrogenases, induces a blue shift of 10–15 nm (43–50) (Figure 2).

In addition to spectral shifts, fluorescence lifetime—the time elapsed between excitation and emission—can be used to assess the protein binding state of NAD(P)H. Although differences in NAD(P)H fluorescence lifetime are commonly attributed to protein binding state, fluorescence lifetime is also sensitive to changes in pH, viscosity, and temperature (Supplemental Table 1). Free NADH in solution exhibits a biexponential lifetime decay, which is attributed to folded versus extended molecular conformations, with characteristic lifetimes of 0.25 and 0.7 ns, leading to a mean lifetime of approximately 0.4 ns (44, 46, 48). The lifetime of bound NAD(P)H depends highly on the substrate. For example, NADH bound to malate dehydrogenase (MDH) has been characterized by a biexponential decay with 0.28-ns and 0.96-ns characteristic lifetimes, leading to a mean lifetime of 0.8 ns (46). A similar mean lifetime has been reported for NADH bound to MDH but was attributed to three distinct decay times of ~0.3, 0.79, and 1.69 ns, depending somewhat on the relative ratio of NADH to MDH (51). Fluorescence from NADH bound to L-LDH (L-lactate dehydrogenase) exhibited a multiexponential decay with lifetimes of 0.34, 1.1, and 2.53 ns, yielding a mean lifetime of 1.08 ns (51). Binding to liver aldehyde dehydrogenases has been reported to yield a significantly longer decay, with constants of 1.8 and 4.2 ns; however, these measurements were performed at 1°C, and lifetimes are expected to be longer at lower temperatures (52). Mitochondrial matrix NAD(P)H fluorescence from intact heart mitochondria has been found to exhibit three characteristic pools with lifetimes of 0.4, 1.9, and 5.7 ns (44). The 0.4-ns component was attributed to free NAD(P)H and represented 63% of the NAD(P)H concentration and 23% of the overall NAD(P)H mitochondrial fluorescence. The 1.9-ns component was attributed to NAD(P)H bound to dehydrogenases, representing 30% of the NAD(P)H concentration and 45% of the overall fluorescence. Finally, the 5.7-ns component was identified as NADH likely associated with complex I of the ETC (44, 45). While it is only 7% of the NAD(P)H concentration, it represents 30% of the NAD(P)H fluorescence signal. While binding of NAD(P)H to dehydrogenases has been typically reported to yield a moderate (1.2- to 2.2-fold) fluorescence quantum yield enhancement (48), binding to complex I led to a 10-fold enhancement (45). The quantum yield of NADH fluorescence has also been shown to increase as the polarity of the solvent/environment increases and as the temperature decreases (52). Thus, while the amount of NAD(P)H that is expected to be in a bound state is less than a quarter of the overall cellular NAD(P)H concentration, the longer lifetime and enhanced quantum yield result in the bound NAD(P)H as the significant contributor to the detected fluorescence. In addition, it has been recently reported that 25–30% of free NAD(P)H may be in a dark state that is attributed to quasi-static self-quenching, a process that was not detected for bound NAD(P)H, leading to further underestimation of free NAD(P)H levels on the basis of lifetime measurements (46).

As indicated earlier, the spectral signatures of NADH and NADPH are identical, since the additional phosphate group is part of the adenine end of the molecule far away from the nicotinamide ring, considered responsible for fluorescence emission. In some studies, changes in the levels of NAD(P)H intensity have been attributed to relative changes in NADPH and NADH concentrations (53). Studies involving genomic modifications that led to significant up- or downregulation of cellular NADPH levels and associated competitive inhibition of NADPH binding have indicated that a longer fluorescence lifetime is associated with bound NADPH compared with bound NADH (36, 54). While changes in the lifetime of the bound NAD(P)H component could serve as a more specific indicator of relative changes in NADH and NADPH concentrations, changes in binding state of a variety of protein complexes can influence the lifetime of bound NAD(P)H, as described in the previous paragraph. Furthermore, the overall levels of NADPH may be quite low in some cellular systems, such as epithelial and mesenchymal stem cells (55, 56). Thus, in several studies, the contributions from NADPH are not considered to significantly affect observed changes or differences in NAD(P)H TPEF signal.

Typically, the NAD^+^/NADH ratio is the focus of investigations into cell redox state. However, NAD^+^ is not fluorescent, and flavin-associated autofluorescence is typically used as a surrogate measurement during optical imaging. Riboflavin (or vitamin B_2_) is the main precursor of flavins in mammalian cells. Flavin mononucleotide is a phosphorylated derivative of riboflavin, to which an adenosine monophosphate group can be added to yield FAD. A small proportion of FAD exists in a free, unbound state, but the majority of FAD autofluorescence comes from FAD moieties within flavoproteins. Three main pools of cellular flavin autofluorescence have been identified (57–59). Nonspecific flavin autofluorescence has the most red-shifted fluorescence emission peak at ~525–530 nm, where it represents approximately 25% of the overall flavin-associated mitochondrial fluorescence (Figure 2). The most significant flavoprotein signal (typically more than 50%) is believed to emanate from lipoamide dehydrogenase (LipDH), with a blue-shifted peak relative to that of free FAD, occurring at 515–520 nm, and a 2.2 times higher quantum yield (Supplemental Table 1). The remaining flavin fluorescence signal has been attributed to electron transfer flavoprotein (ETF), typically associated with transferring reducing equivalents from β oxidation of fatty acids to coenzyme Q in the ETC. The levels of this signal can vary significantly depending on the cell type, with very little contribution expected in brain mitochondria and up to 20–35% of flavin-associated fluorescence in liver and muscle mitochondria (47, 57, 58). The emission peak of ETF is expected in the 485- to 515-nm range and has a 3.5-fold enhanced quantum yield relative to free FAD (58).

Of these three pools of flavin autofluorescence, FAD fluorescence emanating from LipDH is most likely to change in response to metabolic perturbations. Multiple TCA protein complexes contain LipDH and are thus in equilibrium with NAD^+^, because LipDH transfers electrons released during catabolism to produce NADH (53). In fact, the absence of changes in LipDH-associated fluorescence when NAD(P)H fluorescence is altered may provide opportunities to identify changes in the NADPH rather than in the NADH cellular pool, variations that are not easy to distinguish otherwise. The two-photon excitation spectra of free FAD and LipDH-FAD are similar, but not identical; the 850- to 950-nm features are slightly blue shifted on the basis of what would be expected from doubling the wavelength of the one-photon excitation (Figure 2). Interestingly, the excitation peak in the 700- to 750-nm range is more dominant than in the 850- to 950-nm range, a result that is not consistent with the relative height of the one-photon spectra.

The fluorescence lifetime of FAD depends on its binding state, as expected (Supplemental Table 1). While free FAD is typically considered to have a longer lifetime than bound FAD [in contrast to NAD(P)H], a wide range of lifetimes has been reported for both states. Free FAD in water exhibits a biexponential decay, with an ultrashort 7-ps component, which is essentially not detected in most measurements, and a longer component with a lifetime in the 2.2- to 2.7-ns range for temperatures varying between ~40°C and 20°C, respectively (60). Quenching that results from the stacking of the flavin and adenine ring systems of FAD is associated with these differences in lifetimes, with the longer lifetime corresponding to an open conformation. A very small percentage of FAD molecules is expected to be in that open conformation, on the basis of molecular dynamics simulations (60). This is one of the reasons why detected FAD fluorescence levels are typically very low. Flavin fluorescence measurements in heart myocytes revealed significant contributions from free FAD, with a peak emission at 522 nm and a lifetime of 2.47 ns, and a second component attributed to LipDH-FAD, with an emission peak of 504 nm and a biexponential decay characterized by 0.88- and 4.14-ns decay times and relative amplitudes of 35% and 65%, respectively (61). In a different study, free FAD in solution was reported to exhibit a biexponential decay with a mean lifetime of 3.13 ns, attributed to decay constants of 2.57 ns (71% contribution) and 4.42 ns (29%). The fluorescence lifetime of FAD bound to LipDH had a slightly lower mean lifetime of 2.75 ns and exhibited a triexponential decay, with constants corresponding to 268 ps (34%), 2.17 ns (26%), and 5.3 ns (39%) (62). Cardiomyocyte fluorescence emission with ETF-consistent maxima was associated with a mean lifetime of ~1 ns (61). Thus, interpretation of the FAD-associated fluorescence lifetime is more difficult, especially without the additional context of other metabolic information.

## MITOCHONDRIAL ORGANIZATION

While fluorescence intensity and lifetime have been the main sources of optical contrast for characterizing cellular metabolism, more recently, it has become evident that changes in the localization patterns of these signals can also serve as a valuable metric of metabolic function. Isolation of the NAD(P)H fluorescence from the cytosolic and mitochondrial compartments and quantification of changes in each compartment are challenging but feasible. As indicated above, the quantum efficiency of NAD(P)H fluorescence is enhanced from 2- to 10-fold when bound, compared with that of its free state, and NADH concentrations are significantly higher in the mitochondria (34). Thus, it is reasonable to assume that higher intensity intracellular NAD(P)H fluorescence features are associated with mitochondria. Co-staining with MitoTracker^™^ stains has confirmed this assumption in keratinocytes and stem cells (55, 63). In flattened pancreatic islets, for example, the NAD(P)H fluorescence intensity was used to segment mitochondria and highlight the presence of 10-fold higher NAD(P)H levels in the mitochondria versus the cytosol (49). Similarly, significantly higher levels of NAD(P)H autofluorescence are detected in the mitochondria (86% ± 10%) versus the cytosol (14% ± 6%) of nontransformed breast epithelial cells (51).

**Figure 3:**
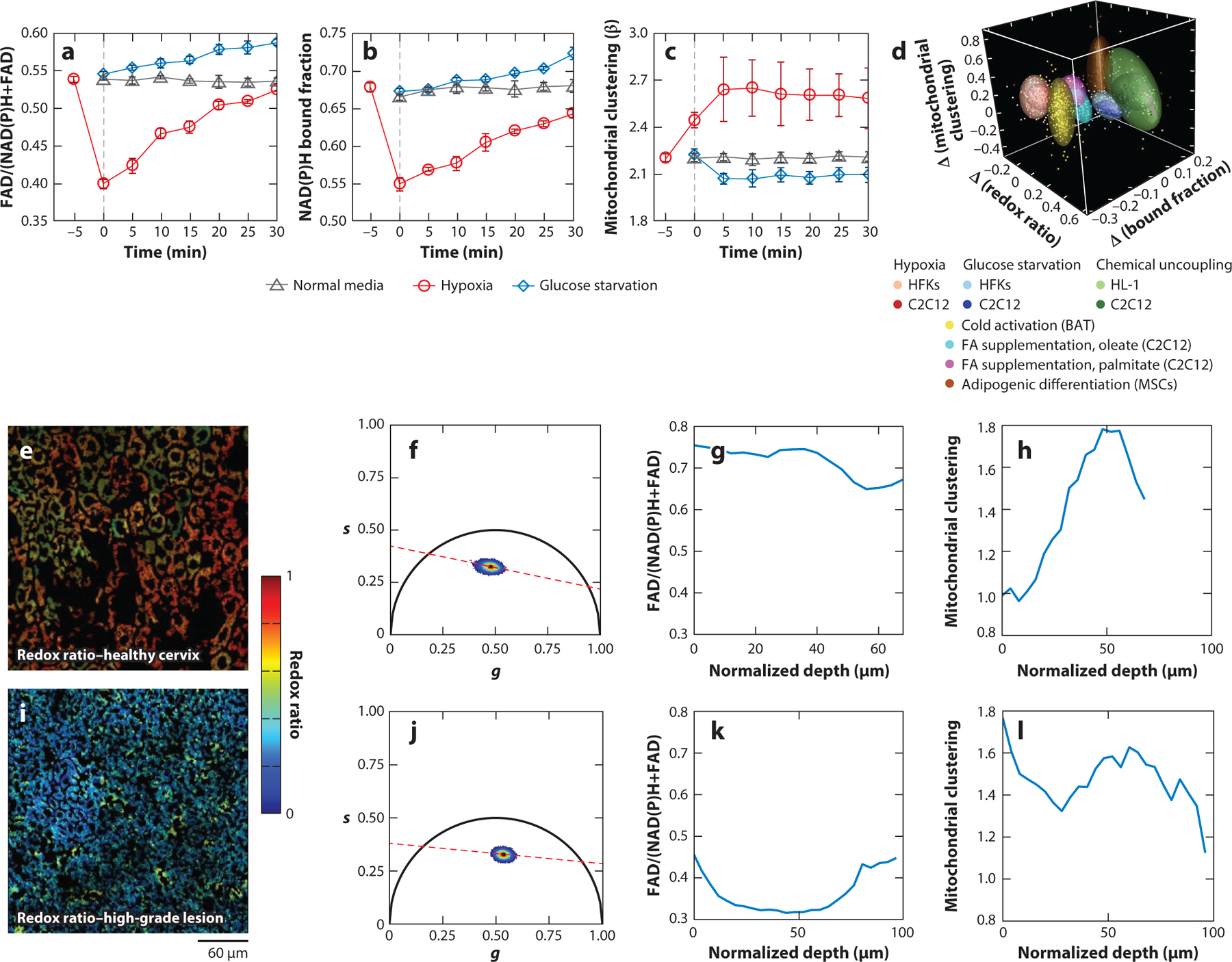
Label-free assessment of metabolic state using two-photon microscopy. (*a*–*d*) Optical monitoring of dynamic metabolic changes induced by hypoxia (enhanced glycolysis) and glucose starvation (enhanced glutaminolysis) compared with untreated primary HFKs. (*a*) Optical redox ratio, (*b*) NAD(P)H bound fraction, and (*c*) mitochondrial clustering exhibit dynamic changes that depend on the nature of the metabolic pathway change. The dynamics of the mitochondrial clustering changes are highly distinct from those of the other two metrics. (*d*) The combination of changes in the optical redox ratio, NAD(P)H bound fraction, and mitochondrial clustering metrics can be used as sensitive and specific biomarkers of key metabolic pathway changes. (*e*–*l*) Sensitivity of optical metabolic readouts to the onset of human cervical precancers as detected in freshly excised human cervical tissues. (*e*,*i*) Representative optical redox ratio coded images, (*f*,*j*) phasors generated from the corresponding lifetime images at 755-nm excitation and 450-nm emission, and (*g*,*h*,*k,l*) depth-dependent variations in (*g*,*k*) the optical redox ratio and (*h*,*l*) mitochondrial clustering across the epithelial depth. Changes in the hues in panels *e* and *i* highlight distinct redox states, as do the locations of the corresponding phasors. The depth dependence of these metrics also yields highly diagnostically useful information (67), demonstrating the importance of high spatial resolution in all three dimensions. Abbreviations: HFK, human foreskin keratinocyte; MSC, mesenchymal stem cell; NADH, reduced nicotinamide adenine dinucleotide; NADPH, reduced nicotinamide adenine dinucleotide phosphate. Panels *a*–*d* adapted with permission from Reference 93 (CC BY-NC 4.0). Figure generated by Varshini Ramanathan with contributions from Christopher Polleys, Tufts University.

We have shown that NAD(P)H intensity patterns can be analyzed via an automated Fourier-based analysis approach to extract a robust and quantitative metric of the organization of mitochondrial networks (63–65). In this approach, cytoplasmic regions are defined and a clone-stamping procedure replicates intracellular patterns in the image background to remove intensity patterns produced by the nuclear and/or cellular border regions. Next, the power spectral density of the 2D Fourier transform of the clone-stamped NAD(P)H images is radially averaged to produce a 1D power spectral density that can be fit for a range of spatial frequencies, *k*, corresponding to subcellular features, such as mitochondria, with an inverse power law, *R*(*k*) = *Ak*^−β^. The exponent (β) is a metric of mitochondrial clustering, with higher values corresponding to more fragmented mitochondria and lower values to more highly networked ones. This metric has been validated with mitochondrial stains, and we have demonstrated its sensitivity to mitochondrial organization changes in a wide range of specimens, from monolayer cultures to in vivo humans (63–65) (Figure 3). We demonstrated, for example, that mitochondrial clustering values assessed from analysis of NAD(P)H TPEF images acquired from human skin in vivo exhibit a reproducible depth-dependent pattern that reflects the distinct metabolic states of the different epithelial layers (63, 66). This depth dependence has been reproduced for mature cervical squamous epithelial tissues (67) (Figure 3). Importantly, we showed that, using this approach, we were able to detect dynamic changes in the mitochondrial organization of human basal layer keratinocytes in response to hypoxia induction in vivo (63), as well as metabolic changes associated with the presence of cancer and vitiligo lesions (63, 66, 67). These Fourier-based approaches typically involve extracting a measure of mitochondrial organization (β) from entire image fields, but more recently, similar approaches that operate in the spatial domain have been adapted to measure mitochondrial clustering on a pixel-by-pixel basis and have been applied in cardiovascular and cancer studies (68, 69). Collectively, a growing body of literature demonstrates that measures of mitochondrial organization from NAD(P)H imaging can complement the functional information derived from fluorescence intensity and lifetime characteristics.

## POTENTIAL CONTRIBUTIONS FROM OTHER ENDOGENOUS FLUOROPHORES

There are several challenges associated with the use of endogenous fluorescence to assess metabolic function. The signals from NAD(P)H and FAD are inherently weak, so there is often a compromise between data acquisition time and signal-to-noise ratio. This issue may be significantly complicated by the presence of other endogenous fluorophores in addition to NAD(P)H and FAD, which may have overlapping fluorescence characteristics. When signals from other endogenous fluorophores are collected, they exhibit spatial and temporal changes within samples that can be mistaken for changes in metabolic cofactor concentrations. Below, we highlight some of the main endogenous cellular fluorophores that could interfere with the robust identification of NAD(P)H and FAD-associated readouts (Figure 2 and Supplemental Table 1).

Carotenoids, which play an important antioxidant role, are potential contributors to cellular autofluorescence. β-carotene and lutein have been shown to exhibit weak fluorescence with a peak near 520 nm when dissolved in hexane, while carotenoid-associated fluorescence in brain tissues had broad features with a maximum near 540 nm (70). Carotenoids with three distinct lifetimes of 0.03, 0.3, and 3.5 ns have been identified in yeast, where fluorescence lifetime was used to assess the abundance of different carotenoids on the basis of growth medium and phase (71). Mean autofluorescence of carotenoids such as lutein and zeaxanthin is in the range of 30–80 ps.

Carotenoids are precursors to vitamin A retinoids, including retinol, retinaldehydes, and retinoic acid (72). Retinaldehydes have a significantly lower fluorescence quantum yield than retinol (72). On the other hand, all-*trans*-retinal and its condensation products, which form during natural aging, are much more fluorescent than retinoids and are considered harmful (72). Di-retinoid-pyridinium-ethanolamine (A2E) is such a condensation product, which is also available commercially as a synthetic compound, and has been characterized in several studies (73). Retinyl palmitate, a main component of retinosomes, is broken down into retinol and palmitic acid, while retinoic acid is derived from retinol. Synthetic retinyl palmitate exhibits a broad peak at ~480–520 nm and a triexponential lifetime decay with an amplitude weighted lifetime of 1.6 ns (73). This is somewhat shorter than the 2.17-ns mean lifetime reported for retinyl palmitate in liposomes (74). Two- and three-photon excited fluorescence of retinosomes is consistent with all-*trans*-retinol solution in ethanol and includes two distinct peaks at 480 and 521 nm of similar intensity. Interestingly, the ~520-nm peak is not as prominent, appearing only as a shoulder in retinol single-photon excited spectra (73, 74). Dimethyl sulfoxide (DMSO)-dissolved A2E spectra exhibit an emission maximum at 650 nm and a biexponential lifetime decay, with constants that depend on wavelength and concentration. For example, at a concentration of 10 mM, the mean lifetime decreased from 0.3 to 0.14 ns when the two-photon excitation wavelength was changed from 750 to 850 nm. Similar (~0.2–0.32 ns) mean lifetimes have been reported by other studies of lipofuscin-associated eye pigments, with small differences depending on the age of the patient’s eyes and the spectral range of detection (498–650 nm versus 560–720 nm, with overall shorter lifetimes at longer wavelengths) (75). Two-photon excited fluorescence lifetime analysis from intact mouse eyes exhibits patterns consistent with the presence of retinyl esters and A2E but are broader, possibly as a result of the impact of the environment and/or additional retinal condensation products (73). A2E is considered to be an important contributor to the lipofuscin-associated autofluorescence of the retinal pigment epithelium (RPE), but there are studies that indicate that the similarity in the spectral emission from A2E and lipofuscin granules may be coincidental (76). Even though lipofuscin has been studied extensively in the context of the RPE, it is found in many types of postmitotic cells, including neurons and myocytes (77). It is considered to be a strongly fluorescent, indigestible mixture of cross-linked proteins and lipids, often associated with lysosomes. The composition of lipofuscin granules can vary significantly, with retinal lipofuscin including a very small percentage of protein (~2%) compared with that of other lipofuscin granules, which typically contain 30–70% cross-linked proteins (77, 78). Spectrofluorometric studies of 1,4-dihydropyridines, Schiff base polymers, and malondialdehyde polymers, which are assumed to be some of the main contributors of lipofuscin autofluorescence, exhibit significant concentration-dependent changes to their emission spectra; such dependence is thought to provide the basis for a wide variation in lipofuscin-associated fluorescence (79).

For skin or RPE imaging, melanin autofluorescence contributions can also be significant. The single- and two-photon excitation cross-section spectra of melanin are very broad. Two-photon excitation of melanin fluorescence in human skin exhibited a maximum at 720–760 nm and decayed ~3-fold at 880 nm (80). The emission of synthetic melanin is dependent on the solvent, with broader features present when melanin is dissolved in DMSO as opposed to water; two-photon emission spectra appear broader and red shifted compared with one-photon spectra, especially in DMSO (81). Pheomelanin and eumelanin have been shown to have distinct emission and lifetime characteristics. Spectra isolated from hair indicate an emission peak at ~625 nm for pheomelanin (found in red hair) and at ~650 nm for eumelanin (found in black hair) (82). Phasor analysis of fluorescence lifetime data indicates multiexponential decays for both types of melanin, with eumelanin characterized by shorter lifetimes (82). These findings are consistent with studies using synthetic melanin that reported a triexponential fluorescence decay with lifetimes corresponding to 200 ps, 1.5 ns, and 5.8 ns (81). Biexponential melanin fluorescence lifetimes have been reported by several studies considering cell cultures and human skin cell populations (83, 84). A short lifetime in the range of 120–140 ps is reported in several studies, along with a longer lived component with lifetimes in the range of 1,076–1,920 ps (83, 84). Interestingly, particularly for eye-related studies, eumelanin and lipofuscin appear to have very similar spectral and lifetime features, especially when measurements are made in biologically relevant samples.

For keratinizing epithelia, lining the skin and portions of the oral cavity, keratin TPEF emission needs to be isolated and removed from analysis involving NAD(P)H and FAD. The keratin emission spectrum in human skin samples depends on the excitation wavelength, with the peak emission wavelength shifting from 465–475 nm to 503 nm when excitation wavelengths change from ~760 to 860 nm (85, 86). Gray hair emission attributed to keratin peaks at ~550 nm when excited at 900 nm, but it is not clear if there may be other fluorophores that contribute (82). Pure keratin TPEF at 750 nm exhibits a monoexponential lifetime decay with a constant of 1.4 ns, which is consistent with lifetimes attributed to keratin from cuticles and hair (87).

In summary, the presence of highly overlapping fluorescence excitation/emission spectra and lifetime profiles of the potential chromophores that contribute to detected cellular signals make robust quantification and interpretation of metabolic function from NAD(P)H and FAD autofluorescence challenging. The dependence of these fluorescence signatures on the fluorophore concentration and molecular microenvironment further complicate this task. The combination of spectral and lifetime image collection yields a more complete characterization of the system and further enhances the potential for understanding the origins of the observed signals. Unfortunately, the weak nature of the fluorescence necessitates long acquisition times, rendering such imaging impractical for most routine experiments. However, combined spectral and lifetime measurements may be used as a basis for the design of more efficient image acquisition schemes at a small number of excitation/emission bands that may still be able to allow fluorophore quantification. The use of ETC inhibitors and uncouplers or other metabolic pathway inhibitors can further validate sensitivity to the detection of signals relevant to metabolic function.

## IDENTIFYING CHANGES TO SPECIFIC METABOLIC PATHWAYS THROUGH NAD(P)H AND FAD IMAGING

Another key challenge associated with metabolic imaging using endogenous fluorescence is establishing the connection between a given change in optical signal and the metabolic pathway perturbation that led to that change. A typical cancer metabolic hallmark is an increase in glycolysis relative to oxidative phosphorylation even under conditions that are not limited by the availability of oxygen, often referred to as the Warburg effect (88). In this case, NADH is produced at a higher rate but not utilized as actively in the mitochondria, leading to an increase in NAD(P)H levels and an overall decrease in the optical redox ratio (Figure 3) (56, 67, 89). We use the definition of the optical redox ratio established by Britton Chance’s group as flavoprotein/(pyridine nucleotide + flavoprotein), which is equivalent to FAD/[NAD(P)H + FAD], for the nomenclature adopted in this review (90). However, NAD(P)H accumulation in the mitochondria, and a corresponding decrease in the optical redox ratio, has also been consistently observed when de novo lipid synthesis is activated, for example, in stem cells undergoing adipogenic differentiation (55, 91–93). In this case, glycolysis occurs at a higher rate to support the production of citrate within the TCA cycle, which is then exported in the cytosol and used for fatty acid synthesis. As glycolysis and pyruvate dehydrogenase complex activity involve reducing NAD^+^ to NADH, there is more NADH generated than is needed to support ATP production. Stimulation of fatty acid oxidation has also been shown to lead to a decrease in the optical redox ratio, since breakdown of free fatty acids into acetyl-CoA in the mitochondria leads to the production of NADH and FADH_2_ (93). Thus, while in all of these cases the optical redox ratio is sensitive to the changes in the mitochondrial redox pool, it lacks specificity. Such needed specificity may be provided by using complementary metabolic readouts, such as those extracted from analysis of lifetime images.

Increased rates of glycolysis are also typically associated with detection of a decrease in the bound fraction of NAD(P)H and the mean lifetime (93–97) (Figure 3). Glycolysis leads to the production of free cytosolic NADH, and increased utilization of the pentose phosphate pathway leads to cytosolic NADPH generation. Additionally, biosynthetic pathways involving pyruvate dehydrogenase or complexes involved in the TCA cycle will produce free NADH within the mitochondria. Thus, we anticipate that the overall levels of bound NAD(P)H will decrease relative to free NAD(P)H levels in cancer and other highly proliferative cells. Since the bound NAD(P)H lifetime is higher than that of free NAD(P)H, the decrease in NAD(P)H bound fraction also typically leads to an overall decrease in the mean lifetime. Interestingly, a decrease in the optical redox ratio is not always associated with a decrease in the NAD(P)H bound fraction. In the case of enhanced fatty acid synthesis, for instance, the NAD(P)H bound fraction is increased (93). Thus, the increased amounts of NADH produced to support the enhanced citrate needs remain bound in coenzymes to drive biosynthesis and energy production. So, the observation of a lower redox ratio in combination with a decrease in the NAD(P)H bound fraction may be an indicator of enhanced glycolysis, while a lower redox ratio accompanied by an increase in the NAD(P)H bound fraction may be associated with enhanced fatty synthesis. This work demonstrates the potential to improve the specificity of NAD(P)H and FAD fluorescence to different metabolic pathways through a combination of fluorescence metrics. In fact, we have shown that the combined use of the optical redox ratio, NAD(P)H bound fraction, and mitochondrial clustering confer high sensitivity and specificity to the changes induced by hypoxia (i.e., enhanced glycolysis), glucose starvation (enhanced glutaminolysis), chemical and cold-activation-induced uncoupling, fatty acid supplementation (enhanced fatty acid oxidation), and adipogenic differentiation (fatty acid synthesis) (93) (Figure 3). The accuracy of classifying a certain combination of changes in the three optical metabolic metrics increased from ~70% when the most diagnostically useful single metric was used [NAD(P)H bound fraction] to ~92% when all three metrics were used (93).

Nevertheless, the sensitivity of fluorescence lifetime characteristics to several environmental factors complicates interpretation. For example, a recent study aiming to establish optical metabolic biomarkers of hematopoietic stem cells demonstrated that while these cells have a lower redox ratio than their more differentiated counterparts, they also exhibit increased bound NAD(P)H lifetimes and fractions (98). Interestingly, the increased bound NAD(P)H lifetime in these studies was shown to be attributed primarily to a higher intracellular pH. The increased bound fraction was shown to reflect an increase in the levels of NADH bound to lactate dehydrogenase in the cytosol, which converts pyruvate to lactate. In contrast, a decrease in mean NAD(P)H lifetime has been observed with increasing mitochondrial matrix pH in other studies based on human embryonic kidney cells (99). The use of pH sensors in combination with NAD(P)H fluorescence lifetime measurements can account for the impact of pH on lifetime measurements and aid in their understanding and interpretation (99, 100). Consideration of the FAD fluorescence lifetime characteristics in combination with those of NAD(P)H may provide additional clues regarding the origins of the optical changes, and quantification of endogenous fluorescence from other biomolecules, such as lipofuscin, may yield further insights.

## SAFETY CONSIDERATIONS

Currently used laser safety evaluation standards, such as ANSI Z136.1 (American National Standard for Safe Use of Lasers) and IEC 60825–1 (International Standard for Safety of Laser Products), provide a comprehensive standard database for laser safety evaluation based on maximum permissible exposure (MPE) thresholds for skin and ocular tissues. Such thresholds have been based on assessments of (*a*) photochemical damage induced by direct light absorption resulting in a chemical or biomolecular reaction that may cause significant damage, such as carcinogenesis; (*b*) photothermal damage resulting from a rise in temperature and thermal injury following light absorption; and (*c*) photomechanical damage invoked in tissue ablation studies (101).

In MPM, photochemical damage is typically considered the result of multiphoton absorption events that are similar to the single-photon damage that occurs with UV irradiation (i.e., DNA damage) or excitation of intracellular coenzymes and porphyrins that lead to formation of ROS (102). Thermal mechanical damage is only considered relevant under multiphoton imaging conditions of highly pigmented tissues, such as skin, and is not expected to be significant in other tissues (103). Seminal damage studies performed with Chinese hamster ovary (CHO) cells indicated that clonogenic damage induced by 120-fs pulses delivered at 80 MHz at 780 and 920 nm follows a *P*^2^/τ dependence, where *P* is the average power and τ is the pulse width (104, 105). Such damage was detected with average powers as low as 6 mW; however, irradiation in those studies was performed using a 40X objective with a numerical aperture (NA) of 1.3 and dwell times of 60 μs/pixel. DNA damage of CHO cells was assessed directly by immunofluorescence assays for cyclobutene-pyrimidine dimers (CPDs), which are considered primarily responsible for carcinogenesis induced by UV irradiation (102). These studies were performed over a range of wavelengths between 711 and 780 nm delivered at different peak intensities and pulse widths and revealed a combination of second- and third-order power-dependent processes, with the relative balance depending on the wavelength and intensity. In addition, CPD levels were linearly correlated to the pixel dwell time (i.e., the dose delivered). ROS were considered the main mediator of damage induced by 800-nm, 170-fs pulses delivered to kidney epithelial cells at powers higher than 7 mW (106). Low-density plasmas following ionization and dissociation of water molecules may be responsible for this damage, since the objective used for this study was of high NA = 1.3 (101).

An increase in autofluorescence, sometimes referred to as hyperfluorescence, has been used as an indirect damage metric of femtosecond pulse irradiation of tissue (107–109). It appears to have broad emission and to be more efficiently induced by longer wavelengths, highlighting the importance of the spectral content of the pulses (108). However, this hyperfluorescence was observed following in vivo illumination of mouse mucosal and brain tissues after acquisition of many frames, even though the power used was 18 and 52 mW, respectively (107, 108). Cryosections of multiple other tissues including liver, kidney, gut, lung, heart, muscle, and skin also demonstrated an increase of endogenous fluorescence due to prolonged irradiation; the onset of this phenomenon depended on the tissue type (107). The NA of the objective used for tissue imaging was 0.8–0.85, which was significantly lower than that used for the cell studies. A recent study assessing the safety of two-photon imaging of the RPE using femtosecond pulses delivered at either 80 or 8 MHz for similar average power levels identified photothermal damage as the main source of damage observed before ionization is expected to occur (73). Photothermal damage was also identified and quantitatively modeled as the main cause of cell death in cultured monolayers of HeLa cells irradiated with focused femtosecond pulses (110). Findings indicate that cells are less susceptible to damage when exposed to high temperatures for a short time compared with mild temperatures for longer times. These studies highlight the strong dependence of damage effects on many parameters of laser irradiation and the specimen. A database to guide MPE determinations is lacking for tissue types other than the eye or the skin. Thus, it is difficult to design instruments with clear safety targets. The US Food and Drug Administration recommends assessments on tissue phantoms, cells, ex vivo tissues, in vivo animals, and, if possible, human tissues. Simple comparisons with established MPE standards for skin and ocular tissues are acceptable, but both tissue types include unique highly pigmented cell populations, which likely impact the interactions between light and tissue components that lead to damage. Thus, establishing a database with relevant laser light beam delivery parameters that are shown to not cause damage in different human or human-like tissues would be very useful.

## TOWARD STANDARDIZING NAD(P)H AND FAD IMAGING

As NAD(P)H and FAD imaging has become more widespread, researchers have taken different approaches to setting up and calibrating imaging systems and have used different methods for computing and summarizing data. Appropriate controls, calibration, and/or validation measurements are always useful. With the technology maturing, there is an increased need to establish a consensus among leaders in the field and to standardize various aspects of these metabolic measurements to enhance rigor and reproducibility.

A variety of different excitation wavelengths and emission bands have been used to isolate NAD(P)H and FAD fluorescence, which makes comparisons among studies challenging. FAD emits across a broad spectrum of wavelengths spanning 480–600 nm, with a peak at approximately 500–525 nm, and can be excited using a range of wavelengths from 350 to 475 nm (700–950 nm for TPEF) (43, 111). This makes its isolation from NAD(P)H fluorescence particularly challenging, since NAD(P)H fluorescence has a broad overlapping emission spectrum that ranges from 400–550 nm (peak at 460 nm) and can be excited with wavelengths less than 400 nm (<800 nm for TPEF) (43, 111) (Figure 2). Thus, eliminating FAD fluorescence from NAD(P)H fluorescence acquisition requires an emission filter that blocks photons longer than 480 nm. Eliminating NAD(P)H fluorescence from FAD fluorescence acquisition requires excitation wavelengths that exceed 400 and 800 nm for one-photon and two-photon excitation, respectively. Widespread adoption of a specific set of barrier filter specifications or excitation wavelengths is unlikely and dependent on a number of factors often outside of the control of individual researchers. Furthermore, factors such as laser power, laser pulse width, detector efficiency, detector gain, and pixel dwell time can vary from day to day or from study to study and affect NAD(P)H and FAD fluorescence intensities as well. Therefore, standardizing autofluorescence imaging to enable comparisons across studies in the future will require established methods for calibration or normalization of fluorescence intensity rather than adherence to specific filter or laser specifications.

To correct for different instrument specifications and parameters, researchers have commonly normalized autofluorescence intensity using dyes of known concentrations. Low concentrations of rhodamine B collected under settings identical to NAD(P)H and FAD imaging have been used to calibrate intensity (95, 112). Others have used low concentrations of fluorescein and established transfer functions relating fluorescence intensity to fluorescein concentration across a range of laser powers and detector gains (55, 92, 113). While these approaches can account for day-to-day changes in settings for a single microscope, they do not account for differences among the efficiencies of NAD(P)H and FAD fluorescence collection between different microscopes or filter sets. To enable cross-study comparisons, NAD(P)H and FAD collection must each be calibrated to a known quantity. This has been done by researchers using the Chance redox scanner, in which frozen solutions of known NAD(P)H and FAD concentrations have been used as a standard reference (114). Application of a similar approach to calibrating TPEF images using NAD(P)H and FAD solutions is an important step in the future, even though the relationship between fluorescence intensity and concentration may depend on additional factors, such as changes in quantum yield or the relative concentration of NADPH, as described in previous sections.

Even with intensity calibration, depth-resolved imaging presents challenges in quantifying NAD(P)H or FAD intensity because of the loss of signal due to the scattering properties of tissues. To account for this, Britton Chance originally proposed a convenient descriptor that is largely independent of depth-dependent effects: the optical redox ratio of FAD/[NAD(P)H + FAD] autofluorescence intensities (27, 29, 90). However, the equation for an optical redox ratio varies throughout the literature, with some groups computing FAD/NAD(P)H or NAD(P)H/FAD ratios as well (115). Obviously, the interpretation of the origins of an increase in the optical redox ratio depends highly on whether FAD or NAD(P)H is used in the nominator. Thus, care must be taken to note the redox ratio definition used in a given study if it is of interest to understand the associated metabolic pathway changes. However, there is a clear statistical rationale for selecting a ratio of FAD/[NAD(P)H + FAD] which is bound between 0 and 1. Establishing this bounded ratio helps to eliminate outlier values when FAD or NAD(P)H intensity values may be at or near zero. Furthermore, if NAD(P)H or FAD intensity distributions each follow a normal distribution, a ratio of either FAD/NAD(P)H or NAD(P)H/FAD will produce right-skewed data that deviate from a normal distribution and violate the assumptions of commonly used parametric tests, such as *t* tests or analysis of variance tests (97). The FAD/[NAD(P)H + FAD] ratio maintains a theoretical distribution much closer to the assumed normal distribution.

In addition to different equations for computing a redox ratio, computing average optical redox ratios from individual images or cells has been performed in different ways. One can average the NAD(P)H and FAD fluorescence intensities from a region of interest and then compute a redox ratio from those average values, or alternatively one can compute pixelwise optical redox ratios and average these ratios within the region of interest. The former weights all photons equally regardless of their location within an image, while the latter weights all pixels equally regardless of their intensity. When the NAD(P)H or FAD fluorescence intensity varies pixel to pixel, different redox ratio values will be computed in practice using these two methods. Computing a ratio from NAD(P)H and FAD intensity averages within a region of interest is generally preferred, because it is less prone to errors caused by instrumentation noise or ambient light in image regions of low NAD(P)H or FAD signal. Furthermore, this method is more likely to be proportional to the ratio of fluorophore concentrations, given that it is weighted by photon counts or light intensity. If averages of pixelwise optical redox ratios are computed for a particular application, accurate segmentation of the region of interest is particularly critical.

NAD(P)H and FAD fluorescence lifetime imaging (FLIM) acquisition and analysis protocols also would benefit from standardization in the future. Most groups measure the FLIM instrument response function (IRF) using second-harmonic generating crystals such as urea or β-BaB_2_O_4_ (95, 113, 116), and the IRF is then convolved with the lifetime decay function prior to curve fitting. However, the extracted time constants and fractions can change depending on specific model parameters and fitting approaches. Parameters are available within commercial software to adjust the location of the IRF relative to the rising edge of the lifetime decay (e.g., shift in Becker & Hickl’s SPCImage), to account for excitation light scattering (scatter in SPCImage) and to correct for ambient light or instrumentation noise (offset in SPCImage). However, when possible, these parameters should be fixed to prevent model overfitting, particularly when selecting a multiexponential decay model in NAD(P)H and FAD imaging (117). The problem of overfitting is particularly challenging with the inherently low quantum yields of NAD(P)H and FAD and low photon counts generated during typical FLIM acquisition. Spatial binning can be used to increase photon counts at the expense of decreasing resolution, and temporal binning has also been found to offer improvements in accuracy (118). Generally, total photon counts of at least 5,000 and decay peaks of at least 100 photons should be targeted for accurate NAD(P)H or FAD FLIM analysis (117). As is the case with optical redox ratio calculations, pixelwise FLIM parameters must be averaged within accurately segmented regions of interest.

In addition to standardizing NAD(P)H and FAD image acquisition and analysis, direct comparisons with biochemical and physiological outcomes are critical to validating the method and evaluating safety. We have previously performed direct comparisons between autofluorescence intensities and intracellular cofactor concentrations as measured through liquid chromatography–mass spectrometry (LC-MS) (55, 56). Specifically, these studies involving differentiating mesenchymal stem cells and human foreskin keratinocytes each demonstrated a strong correlation between the ratios of NAD^+^/(NADH + NAD^+^) concentrations and FAD/(NADH + FAD) autofluorescence. The optical redox ratio has also been benchmarked against metabolic flux analysis, which showed that the redox ratio fluctuation in response to mitochondrial inhibitors and uncouplers matches oxygen consumption rate changes (119). However, as discussed in previous sections, the optical redox ratio is sensitive to fluctuations in catabolic pathways that reduce NAD^+^ in addition to oxidation of NADH by the ETC. Some of the earliest work involving NADH fluorescence has shown its association with increased glycolysis (120), and the optical redox ratio has a strong negative correlation with markers of cell proliferation within the skin epithelium (113, 121). Expanded studies, similar to those described by Liu et al. (93), looking at the relationship between the optical redox ratio and basic mitochondrial and cell functions are needed to further our understanding of this source of contrast and validate its broader use.

## ADDING OTHER SOURCES OF CONTRAST TO NAD(P)H AND FAD IMAGING

Traditionally, NADH and FAD imaging has involved image acquisition using two excitation wavelengths and two emission bands to effectively isolate NAD(P)H and FAD signals from each other. As described in the previous sections, challenges in obtaining relevant metabolic information arise when other endogenous fluorophores may be present within our cells and tissues. Obtaining data from a broader set of excitation or emission wavelengths can aid in identifying and isolating additional fluorophores, particularly when spectral unmixing can be performed. Recently spectral unmixing has been used to separate free NAD(P)H, bound NAD(P)H, FAD, and collagen autofluorescence in aging oocytes (122). When MPM imaging is used, additional sources of contrast such as second-harmonic generation (SHG), third-harmonic generation (THG), SRS, and CARS can provide important context as well. SHG in particular can be used to spatially isolate fibrillar collagen and infer image regions that may contain other autofluorescent extracellular matrix proteins (113, 121). THG and CARS have also been used in cancer and stem cell applications to identify intracellular lipids and provide additional context for changes in cellular redox status (91, 123). Using a photonic crystal fiber source to produce a supercontinuum pulse, Tu et al. (124, 125) were able to rapidly collect TPEF, three-photon excited fluorescence, SHG, THG, and CARS or SRS from tissue sections.

Techniques to measure the vascular supply can also be paired with NAD(P)H and FAD spectroscopy or imaging to provide additional context. Kasischke et al. (126, 127) used NADH fluorescence to investigate astrocyte-neuron interactions and to demarcate oxygen diffusion limits from the microcirculation in the brain. Despite a range of optical tools adapted to measure oxygenated and deoxygenated hemoglobin absorption, there have been limited efforts thus far to combine endogenous sources of fluorescence and absorption contrast in imaging platforms. Several groups have combined NAD(P)H imaging with other optical techniques capable of imaging several millimeters into tissue, such as OCT. OCT was paired with simultaneous autofluorescence spectroscopy measurements sensitive to a combination of collagen and NAD(P)H fluorescence as well as hemoglobin absorption and demonstrated in a rat model of ovarian cancer (128). OCT and multispectral NAD(P)H/FAD FLIM have also been paired for simultaneous biochemical and structural characterization of oral cancer (129, 130). Phase-variance OCT was also combined with NAD(P)H FLIM in a multimodal imaging system to assess wound angiogenesis and metabolic responses following a topical treatment (131). Beyond pairing multiple optical imaging modalities to obtain broader metabolic or structural context, there are opportunities to leverage the noninvasive nature of autofluorescence imaging for direct comparisons with high-content assays. Specifically, relating functional metabolic phenotypes as measured by the optical redox ratio or NAD(P)H lifetime outcomes to genetic or protein information available through discovery-based screening platforms such as genomics, transcriptomics, and proteomics techniques can help toward developing and validating optical biomarkers of disease. While some work has been performed to validate NADH, NADPH, and FAD fluorescence measurements with actual concentrations measured through LC-MS (55, 56), additional broader efforts to relate autofluorescence with metabolomics profiling may also aid in understanding the sensitivity of autofluorescence measurements to specific metabolic pathways.

## ACQUIRING DATA FASTER

Although TPEF has emerged as a preferred technology for imaging NAD(P)H and FAD, laser scanning microscopy is inherently time consuming, as data are typically acquired one pixel at a time. Given the low quantum yield of NAD(P)H and FAD, their relatively weak fluorescence intensities often need to be increased through longer pixel dwell times or frame averaging, which typically involves multiple seconds to acquire a single high-contrast NAD(P)H or FAD image. Furthermore, the most common FLIM-based acquisition approach for NAD(P)H and FAD is time-correlated single-photon counting, which can extend NAD(P)H imaging of a single field from seconds to minutes. These requirements present challenges for evaluating mitochondrial dynamics or rapid changes in metabolism, and they also present a significant barrier for clinical adoption of the technology.

Different approaches have been taken to increase NAD(P)H and FAD image acquisition speeds. Speed can be gained by eliminating depth resolution and relying on one-photon UV excitation with a standard epifluorescence microscope, and various groups have explored autofluorescence spectroscopy approaches that eliminate imaging altogether (132–134). To facilitate high-throughput screening, NAD(P)H autofluorescence measurements have also been adapted to a plate reader format (112). To increase the speed of depth-resolved fluorescence microscopy, multifocal, wide-field, and line-scanning MPM approaches have been developed over the last two decades (135–140). However, their applications to in vivo NAD(P)H and FAD imaging have not been robustly evaluated, and some loss of resolution and contrast would be expected in highly scattering tissues.

**Figure 4:**
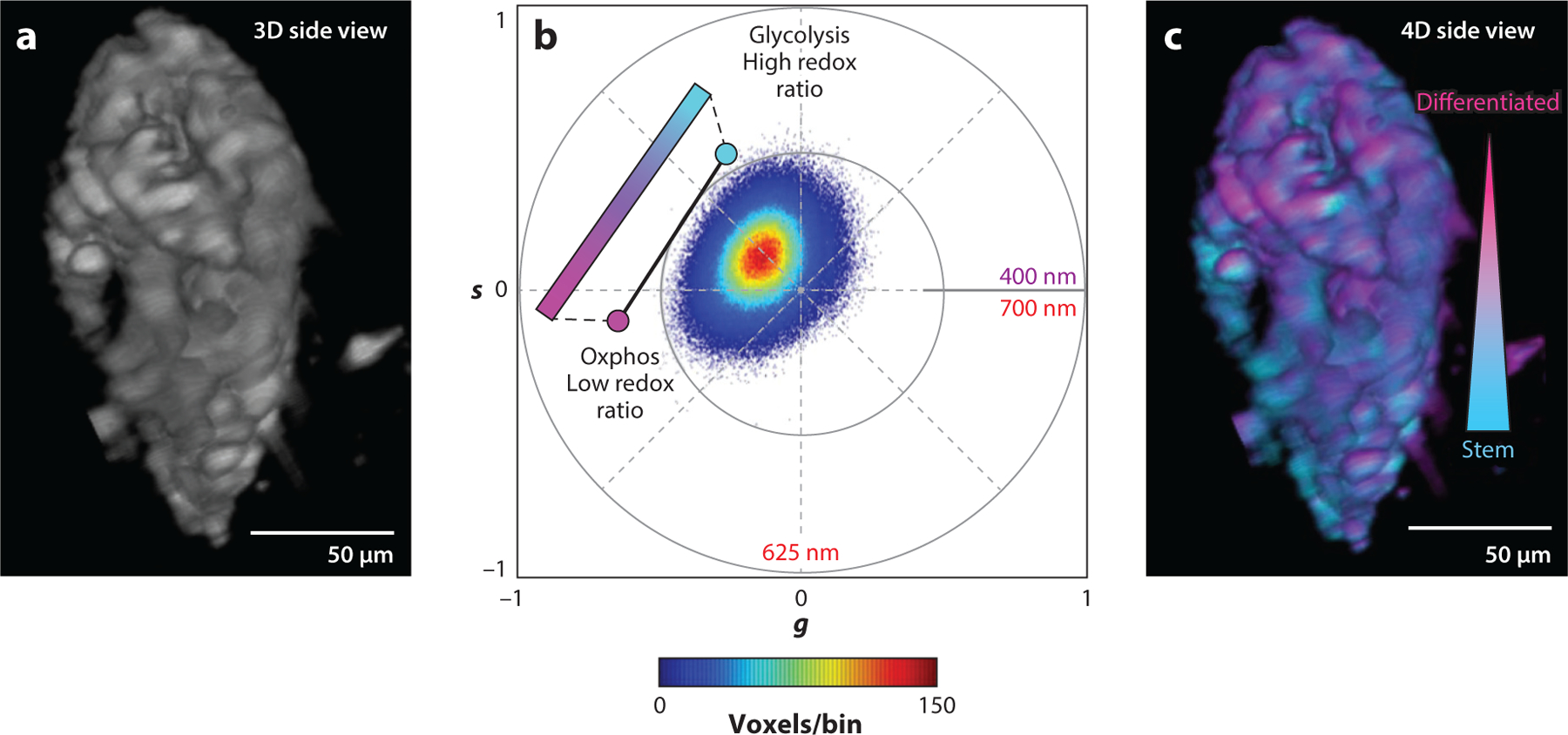
Spectral phasor analysis of metabolism from hyperspectral imaging of mouse colon. (*a*) Maximum intensity projection image of TPEF intensity at 740-nm excitation. (*b*) Spectral phasor plots obtained by sine/cosine transmission filter collection. (*c*) False-color map generated from phasor map to show gradient of stemness within the lumen. Abbreviations: oxphos, oxidative phosphorylation; TPEF, two-photon excited fluorescence. Figure adapted from Reference 149 (CC BY 4.0).

Phasor-based analysis can aid in the rapid acquisition and analysis of NAD(P)H and FAD images by converting photon counts measured across time or wavelength to 2D plots on the basis of the first harmonic of Fourier coefficients (141, 142). Fit-free phasor-based FLIM analysis has been used extensively over the last decade to assess NAD(P)H protein binding on the basis of phasor coordinates and to segment different cell or tissue features (116, 121, 143–146). More recently, spectral phasor analysis has been adapted to NAD(P)H and FAD imaging. Using sine and cosine transmission filters that efficiently collect light representing the spectral phasor coordinates, hyperspectral autofluorescence image data can be acquired very rapidly without the need for sequential scanning across a range of wavelengths (147, 148). Hedde et al. (149) combined this spectral phasor approach with light-sheet microscopy to rapidly collect NAD(P)H and FAD information simultaneously and extract a phasor-based optical redox ratio from live mouse colon tissue (Figure 4). In the future, such an approach can also be adapted to MPM techniques to increase the speed of extracting quantitative metabolic information.

**Figure 5:**
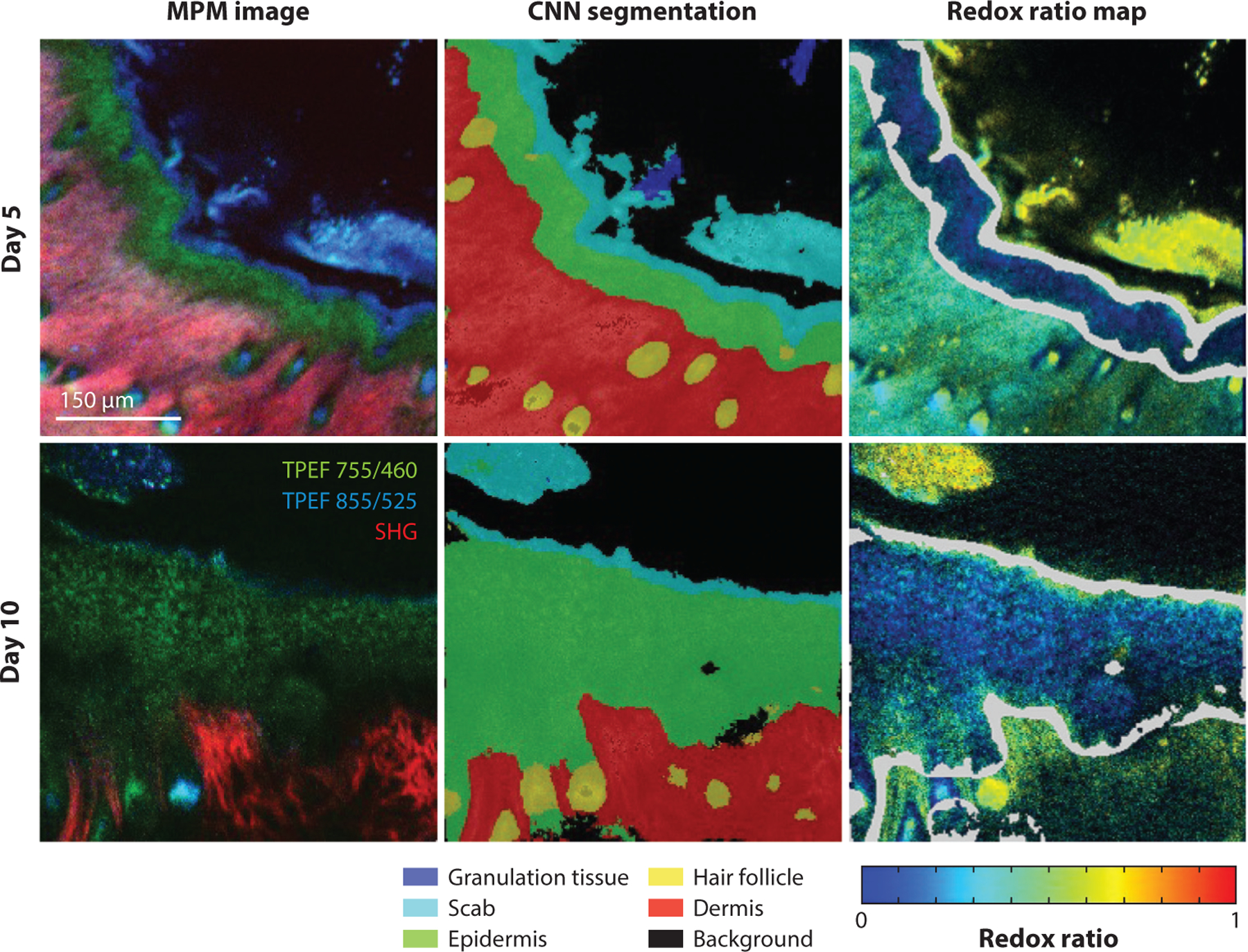
CNN segmentation of in vivo label-free MPM images enabling automated quantification of epithelial redox ratios. Figure adapted from data detailed in Reference 152 that span two time points after creation of a full-thickness skin wound in a mouse model. Abbreviations: CNN, convolutional neural network; MPM, multiphoton microscopy; SHG, second-harmonic generation; TPEF, two-photon excited fluorescence. Figure generated by Dr. Jake Jones, University of Arkansas, and adapted from Reference 152, copyright 2021 Wiley Periodicals LLC.

In addition to advancements in imaging hardware, approaches based on artificial intelligence have the potential to increase the speed of extracting quantitative information from NAD(P)H and FAD images by offering unique solutions to denoising, image generation, image classification, automated segmentation, and virtual staining (150). For label-free MPM specifically, a generative adversarial network architecture was utilized to improve NAD(P)H and FAD image reconstruction and resolution using a commercial microscope with a resonant scanning system (151). Jones et al. (152) developed a multiclass segmentation network for in vivo skin autofluorescence images, enabling rapid quantification of epithelial optical redox ratios (Figure 5). Smith et al. (153) developed a neural network framework to enable rapid fit-free analysis of NAD(P)H FLIM. Continued work in this area, particularly through the use of generative adversarial networks, is likely to continue to increase the speed and robustness of extracting quantitative biomarkers of disease and metabolic health from NAD(P)H and FAD autofluorescence.

## CONCLUSIONS

Label-free MPM imaging of cellular metabolic function offers a combination of unique advantages for the study of metabolism and should become an approach that the broad scientific community considers as a complement to omics-related research as well as other metabolic imaging modalities. Label-free MPM provides a combination of structural and functional metrics, which can be derived in a quantitative way and are highly sensitive to changes in a number of important metabolic pathways. This information can be derived with high temporal and spatial resolution from 3D specimens in a manner that is, in principle, nondestructive and safe. These attributes make this technique ideally suited to characterize the highly dynamic and heterogeneous metabolic interactions within and among cells (Figure 3). Instrumentation and computational advances promise to enhance the speed and information content that can be extracted. Dependence of the signals on the specific environmental conditions and overlapping optical excitation/emission characteristics of relevant fluorophores may pose challenges in the accuracy and the ability to interpret the origins of the optical metrics that are derived. Care should be taken to account for potential artifacts introduced by other chromophores and to perform measurements using parameters that are safe and do not interfere with cell function. Appropriate calibration and validation studies, as well as standardization of these quantitative metabolic readouts, are needed to enhance broader adoption.

## Supplementary Material

Supplementary Table

## Figures and Tables

**Table 1:** Comparison of metabolic imaging modalities

Contrast	Modalities	Advantages	Disadvantages
Exogenous agents	Various clinical and preclinical modalities (e.g., PET, SPECT, optical microscopy)	High specificity and sensitivity, multiple molecular contrast agents available, solutions available from whole-body to high-resolution microscopy imaging	Limited new clinical translational opportunities due to safety concerns, can alter cellular and tissue metabolism, current clinical solutions require ionizing radiation, systems are not portable
Hemoglobin	BOLD MRI	Whole-body imaging possible	High costs, not quantitative, long imaging times, lacks cellular resolution, limited additional metabolic information available without MRS
	DOT/DRS	Low cost, possible to extract oxygenation and total Hb information, can be easily paired with fluorescence spectroscopy	Lacks cellular resolution; highly portable; limited additional biochemical, molecular, or structural information available
	Photoacoustic techniques (e.g., PAT)	Low cost, good balance of resolution and penetration depth	Lacks subcellular resolution, limited additional biochemical or molecular information available
Lipids, metabolites	MRS	Can be paired with MRI, excellent imaging depth possible	High cost, long acquisition times, lacks spatial resolution
	Raman microscopy	Coherent Raman approaches (e.g., SRS and CARS) enable fast acquisition of biochemical information, can be combined with fluorescence microscopy	Long imaging times and poor contrast when optimized for high spectral resolution, high cost for depth-resolved imaging systems
NADH and FAD autofluorescence	Fluorescence spectroscopy	Low cost, fast acquisition times, simple to incorporate into multimodal systems.	No spatial information, challenges in unmixing the contributions of other fluorophores and chromophores, highly portable
	Standard fluorescence microscopy	Low cost, easy to implement, fast acquisition times, subcellular resolution possible	No depth resolution, high potential for UV photobleaching or damage
	Multiphoton microscopy	Efficient collection of signal due to intrinsic depth sectioning, subcellular resolution possible	Imaging depths are limited to 0.5 mm in practice, long acquisition times for 3D imaging
	Fluorescence lifetime	Offers information of NADH protein binding status, can be paired with microscopy and spectroscopy techniques	Long times required for imaging applications, complex data analysis

Abbreviations: BOLD, blood-oxygen-level dependent; CARS, coherent anti-Stokes Raman scattering; DOT, diffuse optical tomography; DRS, diffuse reflectance spectroscopy; FAD, flavin adenine dinucleotide; Hb, hemoglobin; MRI, magnetic resonance imaging; MRS, magnetic resonance spectroscopy; NADH, reduced nicotinamide adenine dinucleotide; PAT, photoacoustic tomography; PET, positron emission tomography; SPECT, single-photon emission computed tomography; SRS, stimulated Raman scattering.
