# Supplementary material for: Label-Free Optical Metabolic Imaging in Cells and Tissues
Source: Annu Rev Biomed Eng. Author manuscript; Available in PMC 2023 Dec 21. (PMC10733979; doi:10.1146/annurev-bioeng-071516-044730)
Supplement: Supplementary Table [file NIHMS1952639-supplement-Supplementary_Table.pdf]

**Supplemental Table 1**: Fluorescence intensity and lifetime characteristics of key endogenous cellular fluorophores.

| Fluorophore | System | Excitation (Max) (nm) | Emission (Max) (nm) | Excitation (nm) | Emission (nm) | Mean Lifetime (ns) | Lifetimes (ns) | Alpha (amplitude) | Fluorescence Contribution | Ref |
|---|---|---|---|---|---|---|---|---|---|---|
| NAD(P)H | In solution | <700 (2P) | ~460 | | | | | | | 1 |
| NADH | In solution (with MDH) | | 457 | 340 | 460 | 0.8 | 0.28, 0.96 | | | 2 |
| NADH | In solution (with MDH) | | ~470 | 355 | >385 | | 0.4, 1.0 | | | 3 |
| NAD(P)H | In solution (with MDH) | | | 740 | 450 | | ~0.3, 0.79, 1.69 | Varies with concentration | | 4 |
| NAD(P)H | In solution (with LDH) | | | 740 | 450 | 1.08 | 0.34, 1.1, 2.53 | Varies with concentration | | 4 |
| NAD(P)H | Porcine heart mitochondria | | ~450 | 335 | 440 | 1.24 | 0.44, 1.88, 5.68 | 0.63, 0.30, 0.07 | 0.23, 0.45, 0.32 | 5 |
| NAD(P)H | Breast cells; mitochondrial | | | 740 | 450 | 1.3 | 0.6, 3.4 | 0.71, 0.29 | ~0.31, ~0.69 | 4 |
| NAD(P)H | Breast cells; cytosolic | | | 740 | 450 | 1.2 | 0.6, 3.3 | 0.75, 0.25 | ~0.31, ~0.69 | 4 |
| NAD(P)H | Human muscle | | 450 | 325 | >400 | | | | | 6 |
| | | | | | | | | | | |
| Free FAD | In solution | <700, ~900 (2P) | ~525 | 730, 900 | | | | | | 1 |
| Free FAD | In solution | | | 450-460 | >520 | | 0.007, 0.1, 0.5 2.7 | 0.83, 0.01, 0.02, 0.14 | | 7 |
| Free FAD | In solution | | | | | 3.13 | 2.57, 4.42 | 0.71, 0.29 | | 8 |
| Free FAD | Rat mitochondria | | 525 | 436 | 470-600 | | | | | 9 |
| Free FAD | Cardiac myocytes [a] | | 522 | | 495-550 | | 2.47 | | | 10 |
| | | | | | | | | | | |
| LipDH | In solution | | | | | 2.75 | 0.268, 2.17, 5.3 | 0.34, 0.26, 0.39 | | 8 |
| LipDH | In solution | <700, ~900 (2P) | ~515 | 730, 900 | | | | | | 1 |
| LipDH | Rat mitochondria | | 520 | 436 | 470-600 | | | | | 9 |
| LipDH | Cardiac myocytes | | 504 | | 495-550 | | 0.88, 4.14 | 0.35, 0.65 | | 10 |
| LipDH | Human muscle | | ~515 | 442, 454 | >450 | | | | | 6 |
| | | | | | | | | | | |
| ETF | Rat mitochondria | | 490 -515 | 436 | 470-600 | | | | | 9 |
| ETF | Human muscle | | 485 | 442, 454 | >450 | | | | | 6 |
| | | | | | | | | | | |
| Lutein | In solution | 443 (1P) | 519 | 350-785 (450) | | | | | | 11 |
| B-carotene | In solution | 450 (1P) | 522 | 350-785 (450) | | | | | | 11 |
| Carotenoids (lutein, zeaxanthin) | In solution | | | 473 | 498-560 | 0.041-0.084 [b] | | | | 12 |
| Carotenoids | Liposomes | | | 467 | 520 | | 0.033, 0.297, 1.112, 3.94 | 0.73,0.10,0.07,0.09 | | 13 |
| Carotenoids | Yeast on agar | | | 467 | 520 | | 0.04, 0.303, 0.7, 3.5 [c] | | | 13 |
| Carotenoid-associated | Human brain (Alzheimer's) | | ~540 | 470 | >500 | | | | | 11 |
| | | | | | | | | | | |
| All-trans retinol | Liposomes | 328 (1P) | 490 | 325 | | | | | | 14 |
| A2E | In solution | | ~650 | 850 | | | Depends on concentration, excitation | | | 15 |
| Retinyl Palmitate | In solution | | 480-520 | 750 | | 1.6 | | | | 15 |
| Retinyl Palmitate | Liposomes | 330 (1P) | 490 | 325 | 495 | | 2.17 | | | 14 |
| Lipofuscin | Human eye | | | 473 | 498-560 | 0.2-0.32 | | | | 12 |
| Lipofuscin | Human brain (Alzheimer's) | | 560 | 470 | >500 | | | | | 11 |
| | | | | | | | | | | |
| Melanin | In solution | | | 800 | | | 0.2, 1.5, 5.8 | Depends on emission wavelength | | 16 |
| Melanin | Melanocytes | | | 760 | | | 0.14, 1.076 | 0.93, 0.07 | | 17 |
| Melanin | Mouse choroid | | ~620 | 850 | | 0.15 | | | | 15 |
| Pheomelanin | Human hair | | | | | | 0.34, 2.3 | >0.8, <0.2 | | 18 |
| Eumelanin | Human hair | | | | | | 0.03,0.8 | >0.8, <0.2 | | 18 |
| Eumelanin | Human hair | | 640-680 | 1000 | | | | | | 19 |
| Pheomelanin | Human hair | | 615-625 | 1000 | | | | | | 19 |
| | | | | | | | | | | |
| Keratin | Solution; cuticles, hair | | | 750 | | | 1.4 | | | 18 |
| Keratin | Keratinocytes | | | 760 | | | 0.445,2.269 | 0.76,0.24 | | 17 |
| Keratin | Human hair | | ~550 | | | | | | | 19 |

[a] The referenced lifetime was acquired from solution FAD but verified using lifetime measurements from the cardiac myocytes.
[b] Depends on detection range, but referenced information cites the given detection range (498-560); this also applies for other measurements that cite this reference.
[c] Only the longest and shortest lifetimes were found to be significant.

**References**


1. Huang S, Heikal AA, Webb WW. 2002. Two-photon fluorescence spectroscopy and microscopy of NAD(P)H and flavoprotein. Biophys J 82:2811-25
2. Cao S, Li H, Liu Y, Wang M, Zhang M, et al. 2020. Dehydrogenase Binding Sites Abolish the Dark Fraction of NADH: Implication for Metabolic Sensing via FLIM. J Phys Chem B 124:6721-7
3. Lakowicz JR, Szmacinski H, Nowaczyk K, Johnson ML. 1992. Fluorescence lifetime imaging of free and protein-bound NADH. Proc Natl Acad Sci U S A 89:1271-5
4. Yu Q, Heikal AA. 2009. Two-photon autofluorescence dynamics imaging reveals sensitivity of intracellular NADH concentration and conformation to cell physiology at the single-cell level. J Photochem Photobiol B 95:46-57
5. Blinova K, Carroll S, Bose S, Smirnov AV, Harvey JJ, et al. 2005. Distribution of mitochondrial NADH fluorescence lifetimes: steady-state kinetics of matrix NADH interactions. Biochemistry 44:2585-94
6. Kunz WS, Kuznetsov AV, Winkler K, Gellerich FN, Neuhof S, Neumann HW. 1994. Measurement of fluorescence changes of NAD(P)H and of fluorescent flavoproteins in saponin-skinned human skeletal muscle fibers. Anal Biochem 216:322-7
7. van den Berg P, Feenstra K, Mark A, HJC B, AJWG V. 2002. Dyamic conformations of flaving adenine dinucleotide: simulated molecular dynamic of the flavin cofactor related to the time-resolved fluorescence characteristics. J Phys Chem B 106:8858-69
8. Heikal AA. 2010. Intracellular coenzymes as natural biomarkers for metabolic activities and mitochondrial anomalies. Biomark Med 4:241-63
9. Kunz WS, Gellerich FN. 1993. Quantification of the content of fluorescent flavoproteins in mitochondria from liver, kidney cortex, skeletal muscle, and brain. Biochem Med Metab Biol 50:103-10
10. Chorvat D, Jr., Chorvatova A. 2006. Spectrally resolved time-correlated single photon counting: a novel approach for characterization of endogenous fluorescence in isolated cardiac myocytes. Eur Biophys J 36:73-83
11. Lochocki B, Boon BDC, Verheul SR, Zada L, Hoozemans JJM, et al. 2021. Multimodal, label-free fluorescence and Raman imaging of amyloid deposits in snap-frozen Alzheimer's disease human brain tissue. Commun Biol 4:474
12. Dysli C, Wolf S, Berezin MY, Sauer L, Hammer M, Zinkernagel MS. 2017. Fluorescence lifetime imaging ophthalmoscopy. Prog Retin Eye Res 60:120-43
13. Vanek M, Mravec F, Szotkowski M, Byrtusova D, Haronikova A, et al. 2018. Fluorescence lifetime imaging of red yeast Cystofilobasidium capitatum during growth. The EuroBiotech Journal 2:114-20
14. Singh AK, Das J. 1998. Liposome encapsulated vitamin A compounds exhibit greater stability and diminished toxicity. Biophysical Chemistry 73:155-62
15. Palczewska G, Boguslawski J, Stremplewski P, Kornaszewski L, Zhang J, et al. 2020. Noninvasive two-photon optical biopsy of retinal fluorophores. Proc Natl Acad Sci U S A 117:22532-43

16. Teuchner K, Ehlert J, Freyer W, Leupold D, Almeyer P, et al. 2000. Fluorescence studies of melanin by stepwise two-photon femtosecond laser excitation. Journal of Fluorescence 10:275-81
17. Dimitrow E, Riemann I, Ehlers A, Koehler MJ, Norgauer J, et al. 2009. Spectral fluorescence lifetime detection and selective melanin imaging by multiphoton laser tomography for melanoma diagnosis. Exp Dermatol 18:509-15
18. Ehlers A, Riemann I, Stark M, Konig K. 2007. Multiphoton fluorescence lifetime imaging of human hair. Microsc Res Tech 70:154-61
19. Krasieva TB, Stringari C, Liu F, Sun CH, Kong Y, et al. 2013. Two-photon excited fluorescence lifetime imaging and spectroscopy of melanins in vitro and in vivo. J Biomed Opt 18:31107
